# Occupational health and safety hazards among solid waste handlers at a selected municipality South Africa

**DOI:** 10.4102/hsag.v27i0.1978

**Published:** 2022-12-09

**Authors:** Shonisani E. Tshivhase, Ntsieni S. Mashau, Takalani Ngobeni, Dorah U. Ramathuba

**Affiliations:** 1 Department of Public Health, Faculty of Health Science, University of Venda, Thohoyandou, South Africa; 2 Department of Advanced Nursing Science, Faculty of Health Science, University of Venda, Thohoyandou, South Africa

**Keywords:** health, municipality, solid waste, health risk, occupational hazards

## Abstract

**Background:**

Solid waste management is one of the most dangerous occupations in the world because of its potential harm from the waste to the environment and the public. Therefore, the protection of human health and the environment is a challenge that all countries are facing.

**Aim:**

The study explored occupational health and safety hazards among waste handlers at a selected municipality in Limpopo Province.

**Setting:**

The study was conducted in one municipality in the Vhembe District.

**Methods:**

The study used a qualitative, explorative and contextual design to explore occupational health and safety hazards among participants. Participants were sampled using purposive sampling. Data were collected through in-depth individual interviews that lasted between 40 min and 45 min. The sampled size comprised 18 participants and was determined by data saturation. Tesch’s open coding was used to analyse data, where the main theme, categories and sub-categories emerged.

**Results:**

The findings revealed the main theme, namely municipal waste handlers experience occupational health and safety hazards. Four categories emerged from the main theme, namely physical, psychosocial, biological and chemical hazards. The categories were further divided into sub-categories such as exposure to extreme weather conditions and musculoskeletal injuries.

**Conclusion:**

Solid waste handlers experienced preventable physical and chemical occupational health and safety hazards that are inherent to their job. It is recommended that workers be provided with adequate suitable protective equipment to reduce the level of occupational health hazards.

**Contribution:**

The findings in this study will help in closing the gaps by the municipality for failing to prioritise working conditions for municipality waste handlers.

## Introduction

Municipal solid waste handlers play a critical role in maintaining the cleanliness of most urban areas. However, solid waste management is hazardous work that exposes waste handlers to infections and serious accidents (Onoja-Alexander et al. 2020). Over the past few decades, developing countries have experienced increased population growth in urban areas because of improved economic development, rural to urban migration and urbanisation. Urbanisation continues to grow at a faster rate across Africa, hence amplifying waste generation. Globally, municipal solid waste generation was expected to increase from 2.01 billion tonnes per annum in 2016 to 3.40 billion in 2050 (Dlamini, Simatele & Serge Kubanza 2019; Kaza et al. 2018). The Department of Environmental Affairs (2018) reported that South Africa is being challenged by the waste crisis as 10% of its waste is recycled, and most landfills will reach their capacity before 2025. This unprecedented waste generation in most developing countries such as Brazil and sub-Saharan Africa requires proper solid waste management (Godfrey et al. 2019; Tomita et al. 2020).

Urban centres in most developing nations struggle to manage solid waste properly because of increased trash creation and poor waste management systems, waste generation and poor waste management methods (Srivastava et al. 2015). The waste generated in the urban centres of most developing nations is collected manually, instead of using hydraulic lifts. Municipal solid waste handlers carry out different duties such as picking up waste, manually loading and emptying refuse bags into vehicles and disposing of waste in landfills. It is a result of these activities and the hazards associated with waste handlers that exposed them to different health and safety hazards (Moussiopoulos, 2017; Thakur, Ganguly & Dhulia, 2018; Lopez-Arquillos et al. 2019).

In India, Jayakrishnan, Jeeja and Bhaskar (2013) found that waste handlers suffered from occupational-related ailments such as eye disorders, injuries and breathing problems. Moreover, Bogale and Tefera (2014) reported high rate of occupational accidents (43.8%) among waste handlers in their Ethiopian study. A study conducted among solid waste handlers at the Beitbridge Town Council and Gweru City in Zimbabwe revealed that municipal waste workers were exposed to biological hazards such as Gram-negative bacteria and fungi in the truck cabins, high prevalence of musculoskeletal disorders, incidents of diarrhoea, viral hepatitis and obstructive and restrictive respiratory disorders (Emiru et al. 2017; Jerie 2016). The high prevalence of occupational health challenges among solid waste handlers in developing countries is attributed to inadequate manpower, budget constraints, shortage of personal protective equipment (PPE) and low level of mechanisation (Bleck & Wettberg 2012; Melaku & Tiruneh 2020).

Solids waste management is the responsibility of local municipalities in South Africa, and it is conducted manually. It is about waste collection from the point of generation to landfill site that included sorting and separation (Sango, Basson & Williams 2016). Just like in any urban area in developing countries, solid waste management in Thulamela Municipality is conducted manually, thus exposing waste handlers to various related health hazards.

There is little information regarding health and safety risks in Thulamela Municipality in spite of the growing evidence of health and safety hazards among waste handlers. To the best of our knowledge, the available literature does not show the extent of health and safety hazards on waste handlers in this municipality. According to the International Labour Organization and the Republic of South Africa constitution, all employees have a right to work in an environment which is free of hazards that are detrimental to their health (Alli 2008; Goldstone 1997). Therefore, the study sought to explore health and safety hazards in Thulamela Municipality as experienced by waste handlers in the Limpopo Province, South Africa.

## Materials and methods

### Study design and setting

The qualitative approach with an exploratory, descriptive and contextual design was used to explore the health and safety hazards among the participants. The study was conducted in one selected municipality in Vhembe District of Limpopo Province, South Africa. The selected municipality is one of the four in the Vhembe District and the largest in the Limpopo Province (StatsSA 2020). It covers a geographical area that is predominantly rural. The area is 25 596 km² with a population of 1 393 949, and it is dry and very hot in summer. About 23.9% of people living in the municipality live below the poverty line and youth unemployment is at 58.3% (StatsSA 2020). The selected municipality is the sole player in waste management in the area. There is one landfill in this municipality, and it is run by a contracted private company. Waste collections are done daily and weekly in household and villages within the municipality and in Central Business District (CBD).

### Study population and sampling

The target population consisted of all municipal waste handlers, including general assistants, participating in the collection, transportation and disposal of waste. Participants aged between 25 and 45 years and who had worked with the municipal waste management for a period of 3 years and more were eligible for the study. The justification for their inclusion in the study was their sufficient experiences of handling of municipal waste as it was assumed that they understood and recalled past waste and environmental events. The sample size comprised 18 participants, and it was determined by data saturation. Follow-up questions were asked in response to the information provided by the participants during the interview.

### Data collection

Data were collected after written permission was approved by the municipal manager. Before data collection, the principal investigator had a meeting with the participants to explain the purpose of the study and to request for permission to conduct the study. Thereafter, willing participants were given the opportunity to choose a convenient time and dates for the interviews between March 2019 and April 2019. Participants were also informed that they had the option to withdraw from the interview at any time. The interviews were conducted in private rooms of the municipality waste management depots. The duration of the interviews was between 40 min and 45 min. This allowed the researcher to ask questions, probe further and obtain clarity on the information shared by the participants. Data were collected using Tshivenda and Xitsonga language, depending on the participants’ language of preference through in-depth individual interviews with the participants. All interviews were directed by the following central question: ‘May you please explain to me the health and safety hazards that you experience in your day-to-day work?’ The participants agreed for audio recording during data collection, and field notes were used to record non-verbal behaviours and thoughts about the interview for future reference and to assist in data analysis. After interviewing Participant Number 13, repetition of information was recorded. However, interviews continued to ensure data saturation that occurred at Participant Number 18. Data collection was stopped at Participant Number 18 because there was no new information being generated from the participants. All interviews were conducted by one of the researchers who were fluent in both Tshivenda and Xitsonga languages.

### Data analysis

All audio-recorded interviews were transcribed verbatim by one of the researchers. Language experts from the University of Venda fluent in Xitsonga, Tshivenda and English translated the transcribed data from Tshivenda and Xitsonga into English. Data were analysed using the eight steps of Tesch’s open coding technique as outlined in Creswell (2011). The researcher read all the written transcripts. A list of all the topics was grouped, and similar topics were arranged into major and unique topics. The generated list was compared with the original data. Shortening of topics as codes was made, and codes were also written next to the segment appropriate to the text. Similar codes were grouped into a theme, categories and sub-categories. The transcripts were further re-coded by an independent coder who was an expert in qualitative research. A consensus meeting with all the authors was conducted to compare whether the theme, categories and sub-categories represented data that were provided by the participants.

### Measures to ensure trustworthiness

The researcher confirmed that the study findings were a true reflection of human experience by ensuring the criteria for trustworthiness, namely credibility, dependability, transferability and confirmability (Anney 2014). Credibility was achieved through prolonged engagement with the participants, whereby the researcher spent relaxed time with the participants during data collection. The interaction leads to the development of good relationships between the researcher and participants. During the 45-min interview, to corroborate what the participants said, the interviewer repeated what the participants would have said back to them. To ensure dependability, an audit trail was maintained by keeping the recording device, and field notes were used to increase reliability during all interviews. To ensure confirmability, a discussion of the results was done with an independent coder. Also, an audit procedure was utilised where details of the interview were audio-recorded and transcribed, and a thick description of all process of the study was provided. Also to ensure transferability, thorough descriptions of all the study procedures ranging from methodology, findings and interview extracts were provided. Moreover, the assistance of an experienced qualitative researcher was sought to code and re-code data.

A thorough description of all the study procedures extending from methodology, results and interview quotations was provided to ensure transferability. Furthermore, the assistance of an experienced qualitative researcher was sought to code and re-code data.

### Ethical considerations

Ethical clearance to conduct this study was obtained from the University of Venda Research Ethics Committee (No. SHS/18/PH/34/0612).

The gatekeeping permission was obtained to conduct the study. Ethical procedures were adhered to throughout the study. Participants remained anonymous and were identified through numbers that could not be traced to real participants. Data obtained from participants were kept confidential and safe. Written consent forms were obtained from participants who knew that their participation was voluntary and that they could opt out of the study at any time. Participants were not coerced nor given tokens of appreciation for taking part in the study.

## Findings

### Socio-demographic characteristic

Participants’ ages ranged from 25 years and above, with the oldest being 45 years, where 10 of them were males. Most participants had 6 years and more working experience as waste handlers and were married, and 11 participants were permanent workers. Five participants were driver operators (Table 1).

**TABLE 1:** Demographic profiles of participants.

Participants	Sex	Age	Marital status	Position	Temporary or permanent	Working experience
Participant 1	Male	45	Married	General Assistant	Permanent worker	20 years
Participant 2	Male	34	Married	Driver operator	Temporal worker	11 years
Participant 3	Female	38	Married	General assistant	Permanent worker	06 years
Participant 4	Female	37	Married	Driver operator	Permanent worker	4 years
Participant 5	Female	33	Single	General Assistant	Temporary worker	03 years
Participant 6	Female	29	Single	General Assistant	Temporary worker	03 years
Participant 7	male	44	Married	Driver operator	Permanent worker	12 years
Participant 8	Male	26	Single	General Assistant	Permanent worker	04 Years
Participant 9	Female	41	Married	General worker	Permanent worker	06 years
Participant 10	Male	36	Married	Driver operator	Permanent worker	06 years
Participant 11	male	30	Single	General Assistant	Temporary worker	04 years
Participant 12	male	35	Married	General employee	Temporary worker	08 years
Participant 13	Male	25	Single	General Assistant	Temporary worker	05 years
Participant 14	Female	37	Married	Driver operator	Permanent worker	06 years
Participant 15	Male	42	Married	General Assistant	Permanent worker	06 Years
Participant 16	Male	45	Single	General Assistant	Permanent worker	04 years
Participant 17	Female	33	Married	General Assistant	Temporary worker	11 months
Participant 18	Female	34	Married	Driver operator	Permanent employee	08 years

*Source:* Ngobeni, T., 2019, ‘Health and safety risks among the Thulamela Municipality waste handlers in the Limpopo Province, South Africa’, Doctoral dissertation, University of Venda, Thohoyandou

A summary of the study findings is shown in Table 2. Main theme, categories and sub-categories emerged from the data.

**TABLE 2:** Summary of findings.

Theme	Categories	Sub-categories
Municipal waste handlers experience occupational health and safety hazards	Physical hazards	Lack of personal protective equipment. Noise Exposure to extreme weather conditions Musculoskeletal injuries
	Psychological hazards	Discrimination Community harassment Lack of training
	Biological hazards	Infectious disease from contaminated waste
	Chemical hazards	Respiratory problems

### Theme: Experiences of municipal waste handlers regarding occupational health and safety hazards

Study findings revealed that participants were exposed to various occupational health and safety hazards during their daily duties. The following categories emerged from the main theme: physical, psychological, biological and chemical hazards.

#### Category 1: Physical hazards

Throughout the interview, participants described how concerned they were about the health risks they are exposed to in their daily duties. A participant said:

‘Oh! We are always at risk, you know what? Anything can happen to us at work.’ (Participant 15, male, 42-years-old)

The following sub-categories emerged from this category: lack of PPE, noise, exposure to extreme weather conditions and musculoskeletal injuries.

**Lack of personal protective equipment:** Participants said they lacked PPE resulting in skin rashes, nose bleeding and eye problems. These views are captured in the responses below:

‘It’s very tough in this field, to tell you the truth, (*quiet for a moment*), sometimes we need to conduct our duty wearing refuse bags plastics on our hands as a replacement of hand gloves. We are only given one pair of gloves and if you lose it, it means that you should steal from one of your colleagues or work without them. What if I got cut accidentally by a bottle or a jab from a syringe while collecting waste?’ (Participant 7, 44-year-old male)

‘Since I came here six years ago, they never gave us enough uniform, we are given uniforms once or twice a year and to get a suitable size, yes, is a serious problem, you can find yourself without a protective clothing that fits you.’ (Participant 3, 38-year-old female)

**Noise:** Participants said they experience high levels of noise when working in busy streets from the big garbage truck or other cars. This might lead to participants having hearing problems.

Responses below support the above observation:

‘The streets where we work are very busy and very noisy, I can’t even hear what my co-worker says, we communicate with signs, if I am not careful, one can be knocked by an oncoming truck.’ (Participant 12, male, 35-years-old)

Another participant added:

‘Where I work, there is too much noise which irritates so much in such a way that it affects our eardrums.’ (Participant 11, male, 30-years-old)

**Exposure to extreme weather conditions:** All the participants reported that they were exposed to extreme weather conditions during their work. They work even when the sun is extremely hot because of tight waste collection schedules. All participants stated that they were putting their health at risk as they were required to go to their working points to collect waste no matter how hot the sun was. Health problems such as dizziness, headaches, skin rash, nose bleeding and flu were some of the mentioned ailments linked to extreme weather conditions.

Below is what participants said regarding extreme weather conditions:

‘As you can see, today it is very hot (looking at the sun). Tell me, don’t you think that working under that sun can put your health at risk?’ (Participant 17, female, 33-years-old)

‘The way it is so hot, it is not normal, we get tired very easily and I might be putting my skin at risk of skin cancer. I feel dizziness sometimes and have extreme nose bleeding.’ (Participant 10, male, 36-years-old)

**Musculoskeletal disorder:** Most participants mentioned that they usually experience body pains, especially back pain, because of handling, lifting and emptying heavy containers and bins inside the trucks and even walking for long distances collecting waste bags. Below is what one participant said:

‘There is something strange about this job, as you are working you won’t feel anything, but once you knock off and you are ready to go home, you will be dragging your legs as an indication that you are very tired, and you need to rest. I sleep like a log every day. My body would be very sore.’ (Participant 1, male, 45-years-old)

#### Category 2: Psychological hazards

Participants in this study expressed psychological challenges resulting from various factors such as being harassed by people around the community when undertaking their daily work. A participant said:

‘Sometimes when one wake up in the morning, thinking about going to work especially if you had encountered a problem the previous day … eish! [*Moving the head from side to side*]. You feel stressed when you think that you do not know how you are going to face the day.’ (Participant 18, female, 34-years-old)

The following sub-categories emerged from this category: community harassment, discrimination and lack of training.

**Community harassment:** Participants said they were harassed verbally by people in the streets when collecting waste. Participants said drunk people swore at them and do not answer back fearing physically attacks.

These sentiments are captured in the responses below:

‘Sometimes when we collect waste, we come across drunken people, they swear at us, some of them threaten to beat us.’ (Participant 9, female, 41-years-old)

Other participants said:

‘What I can say is that when we collect dust bins in their household, some people do not allow us in; instead, we are told: “You are the ones who are thieves and now you want to steal from us.” It hurts us a lot.’ (Participant 15, male, 42-years-old)

**Discrimination:** The participants reported that some of them were permanent employees, while some were on a temporary contract. Temporary employee participants complained that they were not given raincoats to protect themselves during rainy days. Participants said this discrimination made them reluctant to go to work because their seniors ignored their grievances. Below is what one participant said:

‘The other thing that I don’t feel alright about this job is the discrimination within this workplace, when it’s raining, we are not given the raincoats or reflectors, it is only given to the permanent employee workers, we are told to use the refuse plastic bags to cover ourselves.’ (Participant 13, male, 25-years-old)

**Lack of training:** Participants reported that they were not trained on how to use PPE, and this lack of knowledge exposes them to health hazards. Responses below capture this issue:

‘I feel that the municipal as a big organisation should hire a team that can strictly assist us in terms of health and safety education, because we just carrying on with our duties without any knowledge.’ (Participant 4, female, 37-years-old)

Another participant added:

‘Umhh, in this job, honestly speaking there are no workshops or just even advice on how we should carry out our work in a safe manner. You as an individual just have to make sure that you work in such a manner that your health will be protected.’ (Participant 6, female, 29-years-old)

#### Category 3: Biological hazards

During the interview, participants were concerned about the health risks they are exposed to because of spending many hours at the dump site where there are lots of flies and mosquitoes. A participant said:

‘TJo!! working as a waste handler is a dangerous job, we can be easily infected by different diseases.’ (Participant 16, male, 45-years-old)

The following sub-category emanates from this category:

**Infectious disease from the contaminated wastes:** Participants felt unsafe collecting waste without appropriate PPE like hand gloves. There is a possibility of being pricked by the needles and cut by sharp objects such as razor blades, knifes and bottles. Participants said that they are afraid of diseases such as HIV and AIDS. Working without quality hand gloves exposed them to injuries from objects hidden in the solid waste. These views are supported by the responses below:

‘The hand gloves that we are given are not of good quality and protective enough because they are too short, the solid waste might drop on your skin as you carry up the dirt container, this can put your health at risk.’ (Participant 5, female, 33-years-old)

Another participant added:

‘Due to lack of hand gloves, many people here tie refuse bags on their hands in order to protect themselves when collecting wastes, just imagine using a plastic on behalf of the gloves what if you get cut by a sharp bottle or stabbed by a used syringe and get the infections.’ (Participant 2, male, 34-years-old)

#### Category 4: Chemical hazards

Participants also highlighted that they were exposed to different chemical risks. A participant said:

‘Eeh! Working in a smelly waste area is risky my dear [*pause*], … especially if I find myself not wearing mask.’ (Participant 14, female, 37-years-old)

The following sub-category emerged from this category:

**Respiratory problems:** The findings also revealed that participants were not supplied with high-quality PPEs. The provided dust masks were of poor quality as it does not protect participants from bad odours and dust that can lead to respiratory problems. Also, those dust masks were not regularly issued as they should. Moreover, participants were not supplied with full body suit. These views are captured in the following response:

‘As we work for hours while inhaling dust, smelly things. We are indeed putting our health at risk of all diseases which goes along with diseases such as tuberculosis and respiratory diseases.’ (Participant 4, female, 37-years-old)

‘The reason why I say my safety is at risk is that we work here without masks to cover our faces, going around inhaling the smoke from car exhausts and things that we burn at the dumping site. We sweep the roads and pour dirty stuff in the trucks. Where do you think, the dust is going? [*he asked*]. Obvious I am going to inhale it.’ (Participant 8, male, 26-years-old)

## Discussion

The study findings revealed that most participants were from the low economic background and most did not have formal education. These study findings are similar to studies conducted in South Africa and Zimbabwe regarding municipal solid waste management (Jerie 2016; Tshivhase & Mashau 2020). Similar findings were reported, which showed that solid waste handlers in developing countries were generally from poor backgrounds, uneducated and less skilled (Lissah et al. 2020; Melaku & Tiruneh 2020). The waste handlers’ work involves manually picking up heavy garbage, doing door-to-door collection and loading refuse bags and dump containers into an operational truck (Sharma et al. 2020). These can be done manually using physical strength or hydraulic lift.

There were more male participants in the current study likely because waste handling requires a lot of physical strength. Ziaei et al. (2018) also found that most of their participants drawn from African countries were males. Similarly, in Nigeria, Inyang (2007) had only male participants in his study. However, Njoku, Edkpayi and Odiya (2019) had more female participants in their study.

In the current study, participants were exposed to adverse health and safety risks because of the lack of PPE. Therefore, participants reported experiencing headaches, injuries, accident cuts, skin problems and respiratory ailments because of inadequate or lack of PPE. The contributory factor for lack and insufficient PPEs is likely insufficient funds allocated for waste management and collection in the municipality as attested by Ngobeni (2019). Similar findings have been reported by Inyang (2007) and Ncube, Ncube and Voyi (2017) who reported that waste handlers experienced injuries, accidents and work-related diseases because of non-availability and non-use of PPE. The current study findings also concur with those by Melaku and Tiruneh (2020) who reported that municipal waste collectors in most developing countries were exposed to occupational health problems because of lack of PPE. Personal protective equipment provides the first line of defence against hazards but are not effective in preventing injuries and accidents. Participants were only provided with face masks and hand gloves but were not given full body cover. Only permanent employees were given adequate protective clothing. Sharma et al. (2020) also found that temporary waste collectors were not provided with PPEs. Similarly, Wafor and Nwafor (2020) recommended that municipal authorities should provide enough PPE for all employees.

Participants also complained that they were exposed to excessive noise levels from busy streets, heavy garbage trucks and the passing traffic. Thus, some participants experienced hearing disorders because they are not supplied with earmuffs. Moreover, high noise levels expose waste collectors to accidents because they cannot hear warning sounds from the cars. Some waste handlers climb and sit on top of the truck full of garbage, thus exposing themselves to falling from the moving truck. Similar findings were reported among waste handlers in Zimbabwe and the Netherlands and the United Kingdom where noise has been blamed for causing most accidents that occurred among waste handlers (Ncube et al. 2017; Pereira-de-Paiva et al. 2017). Also, some noise from municipal solid waste emanates from glass and metal tins during emptying of metal bins on the metal floor of waste collection vehicles (Suthar, Rayal & Ahada 2016). Mehlum and Aarhus (2020) also reported that occupational noise exposure in a workplace is a cause of noise-induced hearing loss and tinnitus as well as other health outcomes like hypertension, distress and occupational injuries.

Participants indicated exposure to extreme weather conditions such as excessive sun and cold weather conditions that expose them to health problems such as respiratory symptoms, itching eyes, skin rashes and flu-like symptoms. This is because participants are not provided with suitable PPE. They were sometimes compelled to use plastic bags to wrap their hands or use bare hands when collecting waste. Parallel results were also confirmed by several researchers who said solid waste management is an outdoor activity that exposes workers to extreme hot and cold temperatures, thus exposing waste handlers to health problems such as dizziness, skin rashes, asthma and skin cancer if protective equipment were not provided (Inyang 2007; Ncube et al. 2017).

Participants mentioned that they suffer from body pains, and most of them complained about backaches because of lifting heavy containers and walking for long distances when collecting waste. Municipal garbage collection is done manually. Similar findings were reported from different studies done on municipal waste handlers who complained about musculoskeletal symptoms such as backache, shoulder ache, wrist pain and joint problems (Bulduk 2019; Zakaria et al. 2017).

Solid waste management is a labour-intensive occupation that requires strength, which involves carrying, lifting, sorting and loading waste into collection vehicles. There is a need to introduce a hydraulic system for removing waste that can be used in garbage trucks to load, *lift* and transport both solid and liquid types of waste. A hydraulic system makes it easier for garbage trucks to load, lift and transport both solid and liquid types of waste (Pires et al. 2019). Most municipalities in developing nations have faced economic challenges; thus, there is a need to develop low-cost measures such as training waste handlers on correct posture (Ncube et al. 2017).

Participants complained of community harassment and discrimination at work as some of the psychological stressors. Participants were concerned that temporary waste handlers were not given protective equipment indicating that managers were not treating participants equally. Similarly, Pereira-de-Paiva et al. (2017) found that municipal waste handlers suffer from historical discrimination because of the low nature of their work. The study further revealed that waste handlers experience physical violence, verbal abuse and spitting from members of the public.

Most municipal waste handlers globally are looked down upon and suffer from emotional abuse, discrimination and harassment from the society (Melaku & Tiruneh 2020; Pereira-de-Paiva et al. 2017). The discrimination experienced by municipal waste workers creates unnecessary stress that leads to low job satisfaction. In Ghana, domestic solid waste handlers experience discrimination from the public, which led to stress, isolation and eventually to low work ethics and productivity. This calls for firm public education to sensitise the public about solid waste handlers, and municipal authorities should also craft anti-discriminatory bylaws to protect solid waste handlers from discrimination (Jeong, Lee & Lee 2016; Lissah et al. 2020).

Furthermore, participants complained of poor relationship with management as a psychological stressor at their workplace. The temporary solid waste workers complained that they were not being given PPE, and hence they were stressed about their working conditions. Ghana and Iran have also reported about poor relationships between solid waste handlers and managers, and this has been linked to poor mental health outcomes such as depression, stress and burnout (Bleck & Wettberg 2012; Lissah et al. 2020). Several researchers from Nigeria, Thailand and South Africa also point out that waste handlers are injured and suffer from bacterial or viral diseases because they do not have proper PPE. Workers suffer from health risks such as dermatitis, cuts, burns, hepatitis, respiratory ailments and bites from snakes or mosquitoes (Made et al. 2020; Ncube et al. 2017).

## Conclusion

Solid waste handlers in the Thulamela Municipality experienced various health and safety hazards because of the nature of their work. Therefore, waste handlers are at risk of physical injury because of working without appropriate PPE. The findings further exposed that participants were suffering from emotional hazards such as harassment and discrimination by their supervisors. They were also exposed to ergonomic hazards because of lifting heavy objects. Participants felt that they were at risk of suffering from musculoskeletal disorders.

### Recommendations

It is recommended that municipal waste handlers be provided with suitable PPE and that they be encouraged to put on their PPE while at work. The use of PPE will significantly lessen the risk of occupational injuries and diseases. To improve the health and safety condition for municipal waste handlers, the municipality must issue appropriate PPE. The municipality should issue overalls and work suits, which are highly visible to reduce the risk of vehicle accidents. The management should establish an occupational health surveillance programme for all employees in the municipality.

### Limitations

The study was only conducted in a selected municipality of the Vhembe District. Therefore, the results cannot be generalised to the rest of the Limpopo Province. Moreover, the study was restricted to waste handlers under Thulamela Municipality who have been working as waste handlers for more than 3 years. Further research should be done on waste handlers using quantitative methods to cover most waste handlers in the district.
